# Advancing Public Health Through Internationally Coordinated Medical Device Registries: Comment on "Quality and Utility of European Cardiovascular and Orthopaedic Registries for the Regulatory Evaluation of Medical Device Safety and Performance Across the Implant Lifecycle: A Systematic Review"

**DOI:** 10.34172/ijhpm.9383

**Published:** 2026-02-03

**Authors:** Herbert Mauch

**Affiliations:** Cochlear AG, Basel, Switzerland.

**Keywords:** Medical Device Registries, Real-World Evidence (RWE), International Coordination, Academic Societies, Post-Market Surveillance

## Abstract

This commentary draws on findings from Hoogervorst et al1 to underscore the urgent need for internationally coordinated medical device registries, addressing the fragmentation and inconsistency currently limiting their utility in Europe. It advocates for registries governed by academic specialty societies to ensure scientific integrity, transparency, and clinical relevance. Such registries can significantly enhance post-market surveillance, support regulatory compliance and accelerate real-world evidence (RWE) generation. The importance of standardized data collection, regular outcome reporting, and contributor recognition to foster engagement and improve data quality is highlighted. By complementing randomized controlled trials (RCTs), registries can detect rare adverse events, inform clinical guidelines and drive innovation. Actionable recommendations for governance, data harmonization and interoperability are given, emphasizing that now is the time for academic societies to lead this transformation for the benefit of patients and healthcare systems globally.

## Introduction

 The systematic review by Hoogervorst et al^1^ provides a timely and critical evaluation of the current state of European cardiovascular and orthopaedic medical device registries. Their findings reveal a fragmented landscape, where the lack of standardization, transparency and international coordination significantly hampers the potential of registries to serve as reliable sources of real-world evidence (RWE). This is particularly concerning in light of the European Union Medical Device Regulation (EU MDR 2017/745), which mandates robust Post-Market Clinical Follow-up to ensure the ongoing safety and performance of medical devices. Registries, when well-structured and governed, can play a pivotal role in fulfilling these regulatory requirements by systematically collecting and analyzing data from real-world clinical settings. However, the current heterogeneity in registry practices across Europe limits their utility not only for regulatory oversight but also for advancing clinical research and improving patient outcomes.

 This commentary argues for the establishment of internationally coordinated medical device registries, ideally under the stewardship of academic specialty societies. By harmonizing data elements, standardizing methodologies and fostering transparency, such registries can transform the landscape of medical device surveillance and RWE generation. Well-designed registries complement randomized controlled trials (RCTs) by providing insights into long-term device performance, identifying rare adverse events and informing evidence-based clinical guidelines. International registries can significantly shorten the time from clinical observation to actionable insight by enabling rapid data aggregation and analysis. For instance, the Australian Orthopaedic Association National Joint Replacement Registry’s early identification of high revision rates in metal-on-metal hip implants led to market withdrawal well before similar findings emerged from clinical trials.^2^ Similarly, the Gauging coronary Healing with biOresorbable Scaffolding plaTforms in EUrope (GHOST-EU) registry provided early evidence on the safety of bioresorbable vascular scaffolds, influencing clinical practice and regulatory decisions.^3^ These examples underscore the potential of registries to serve as early warning systems and catalysts for evidence-based innovation.

## Case for International Coordination

 To address the limitations identified by Hoogervorst et al,^1^ there is a pressing need to establish internationally coordinated medical device registries. Such coordination would enable harmonization of data collection methods, outcome definitions and reporting standards, thereby enhancing the comparability and utility of registry data. International collaboration can also facilitate the pooling of data across countries, increasing statistical power and enabling more robust analyses of device performance and safety. Moreover, coordinated registries can streamline post-market surveillance efforts and foster innovation by providing timely feedback to manufacturers and clinicians.

###  Fragmentation Undermines Utility

 Hoogervorst et al^1^ found that only 33% of quality items were reported by cardiovascular registries and 60% by orthopaedic registries, with wide heterogeneity in data completeness, outcome definitions, and follow-up durations. This fragmentation impedes data pooling, benchmarking, and regulatory decision-making. International coordination can address this by harmonizing data elements and definitions, standardizing follow-up intervals, and promoting shared governance and data access frameworks. The International Medical Device Regulators Forum^4^ has already laid out principles for registry quality, including coverage, completeness, accuracy and reliability.

###  Academic Societies as Stewards

 Academic specialty societies are ideally positioned to lead the development and governance of international registries. Their deep clinical expertise, commitment to scientific integrity, independence and established networks across institutions and countries make them credible and effective stewards. For example, the European Society of Cardiology’s EuroHeart initiative has successfully developed standardized datasets for acute coronary syndromes and heart failure, demonstrating the feasibility and impact of such efforts.^5,6^ Similarly, the International Society of Arthroplasty Registries (ISAR)^7^ has played a key role in promoting global benchmarking standards and implant libraries, facilitating cross-registry comparisons and quality improvement initiatives. By taking ownership of registries, academic societies can ensure that data collection aligns with clinical priorities, supports evidence-based practice and contributes to continuous learning and improvement.

###  Potential Barriers

 Despite their ideal positioning, academic societies have not widely embraced medical device registry governance due to significant financial, organizational, and political barriers. Financially, establishing comprehensive registries requires substantial investment in infrastructure and data management systems that exceed traditional budgets reliant on membership fees and conference revenues. Organizationally, societies lack dedicated operational capacity and regulatory expertise for complex data systems and international coordination, while volunteer-based governance structures with rotating leadership struggle to sustain long-term initiatives requiring consistent stewardship. Politically, academic societies must navigate competing interests among industry sponsors, regulators, and clinical constituencies while managing concerns about neutrality and potential exposure of performance variations that create professional and legal risks.

###  Sustainable Funding Models

 Sustainable funding for academic society-governed registries might require hybrid approaches combining public funding, industry contributions, and user fees with strict governance preventing conflicts of interest. Public funding could provide foundational support for registry infrastructure, recognizing registries as public health goods essential for post-market surveillance. Long-term commitments spanning five to ten years enable strategic planning and robust infrastructure investment. Industry contributions through annual fees proportional to market share could supplement public funding via arm’s-length arrangements preventing influence over data collection, analysis, or publication. Industry benefits from aggregated anonymized data for surveillance and product development might justifying such financial contributions. Tiered user fees from participating institutions could provide additional revenue through value-added services like benchmarking reports and quality improvement feedback. All funding sources and governance arrangements should be publicly disclosed annually, with independent oversight committees reviewing potential conflicts.

###  Legal and Data Protection Framework

 International registry coordination faces significant legal challenges in requiring robust frameworks to protect patient privacy while enabling cross-border data pooling. European General Data Protection Regulation (GDPR) compliance requires clear legal bases for processing (typically “public interest” under Article 6(1)(e)), Data Protection Impact Assessments, and data minimization principles collecting only necessary elements. Cross-border transfers within the EU are facilitated by GDPR provisions, while third-country transfers require additional safeguards like standard contractual clauses. International registries should establish Data Processing Agreements defining roles and security requirements. Pseudonymization, encryption, unique device identifiers, and secure data enclaves should protect patient identities while enabling data linkage and authorized research access. Legal frameworks must address liability issues, providing academic societies protection from litigation arising from data breaches or adverse findings. Public health immunity provisions similar to adverse event reporting systems could extend to registry operations, with clear terms of use defining stakeholder rights and obligations.

## Publishing Outcomes and Recognizing Contributors

###  Transparency and Accountability

 Transparency in reporting is essential for building trust in registry data and ensuring accountability among stakeholders. Yet, as Hoogervorst et al^1^ highlight, only a small fraction of registries report on outlier detection procedures and even fewer make these results publicly available. This lack of transparency undermines the credibility of registries and limits their impact on clinical practice and policy. To address this, international registries should commit to publishing regular outcome reports in peer-reviewed journals and public platforms. These reports should include benchmarking data, risk-adjusted performance metrics and qualitative descriptions of respective outliers. Such practices not only enhance the scientific value of registries but also provide actionable insights for clinicians, regulators, and manufacturers. Examples from the UK National Joint Registry (NJR) and the Swedish Coronary Angiography and Angioplasty Registry (SCAAR) illustrate how regular, transparent reporting can drive improvements in device safety and patient outcomes.^8,9^

###  Balancing Transparency With Commercial Interests

 While transparency is essential for patient safety, academic societies must balance this with legitimate commercial concerns through tiered data access policies. Public reporting should focus on aggregated device-class outcomes informing clinical decisions and regulatory oversight without exposing proprietary details or creating competitive disadvantages. Device-specific commercially sensitive data can be shared selectively with manufacturers, regulators, and researchers under strict confidentiality agreements. Manufacturers benefit from accessing high-quality real-world performance data for product improvements and regulatory submissions.

###  Acknowledging Contributors

 Sustained participation in registries requires significant time and effort from clinicians and healthcare institutions. To maintain engagement and ensure high data quality, it is essential to recognize and reward contributors. This can be achieved through authorship opportunities, acknowledgments in publications and benchmarking that qualifies clinical excellence. Such recognition not only motivates continued participation but also fosters a culture of transparency and continuous improvement.

## Registries as Gold Mines for Real-World Evidence

###  Complementing Clinical Trials

 While RCTs remain the gold standard for evaluating the efficacy of medical interventions, they often have limitations in terms of generalizability, duration, and sample size. Registries complement RCTs by capturing data from broader, more diverse patient populations over extended periods. This enables the detection of rare adverse events, assessment of long-term outcomes and evaluation of device performance in real-world settings. Moreover, registry data can inform the design of future trials, support adaptive regulatory pathways and provide evidence for health technology assessments and reimbursement decisions.^10,11^

###  Accelerating Evidence Generation

 Timely access to high-quality data is critical for identifying safety signals, informing clinical guidelines and supporting regulatory actions. International registries can significantly shorten the time from clinical observation to actionable insight by enabling rapid data aggregation and analysis, as demonstrated by the Australian Orthopaedic Association National Joint Replacement Registry.

###  Global Perspectives

 Beyond European registries, international examples demonstrate diverse implementation approaches. The American College of Cardiology’s National Cardiovascular Data Registry (NCDR)^12^ collects data from over 2400 hospitals, showing how professional societies can drive voluntary participation through benchmarking reports, quality improvement resources, and regulatory alignment. The Society of Thoracic Surgeons National Database similarly demonstrates specialty society leadership driving quality improvement while meeting institutional performance measurement needs. Japan’s National Clinical Database (NCD),^13^ established in 2011, covers 95% of surgical procedures at over 4000 hospitals, achieving remarkable coverage through professional society leadership, integration with board certification requirements, and government support. These international examples illustrate that despite differing contexts, fundamental success factors remain consistent: strong professional society leadership, clear participant value propositions, integration with existing professional infrastructure, and aligned incentives across individual, institutional, and societal interests.

## Recommendations for Implementation

 To realize the full potential of internationally coordinated registries, a multi-faceted implementation strategy is required. First, governance structures should be established under the leadership of academic societies, with representation from clinicians, regulators, patients and industry. Second, core data sets and quality metrics should be defined based on consensus frameworks and aligned with regulatory requirements. Third, participation in registries should be mandated or incentivized and data completeness should be monitored and reported. Fourth, registries should commit to regular reporting and recognize contributors through transparent authorship and acknowledgment policies as well as practical incentives including benchmarking reports, quality improvement feedback, and continuing medical education credits. Finally, interoperability and data linkage should be facilitated through the use of standardized terminologies, unique device identifiers and integration with electronic health records. These steps will ensure that registries are not only scientifically robust but also operationally sustainable and clinically impactful.

## Conclusion

 In conclusion, the systematic review by Hoogervorst et al^1^ highlights both the challenges and opportunities in leveraging medical device registries for regulatory and clinical purposes. By transforming fragmented national efforts into internationally coordinated, academically led registries with a clear mission to publish outcomes and recognize contributors the full potential of RWE could be unlocked. While significant barriers exist, including financial constraints, organizational capacity limitations, legal complexities and competing stakeholder interests, these challenges might be overcome through sustainable funding models, robust legal frameworks, transparent governance and multifaceted contributor incentives. Such registries will not only support regulatory compliance and clinical decision-making but also drive innovation, improve patient outcomes and enhance the overall efficiency of healthcare systems. The time to act is now and academic societies must take the lead in shaping the future of medical device surveillance and evidence generation.

## Disclosure of artificial intelligence (AI) use

 AI has been used to support conceptional research, check grammar and improve overall manuscript clarity.

## Ethical issues

 Not applicable.

## Conflicts of interest

 Author declares that he has no conflicts of interest.

## Disclaimer

 Views expressed in this commentary solely represent my personal views based on my professional experience. They are independent of my current role at Cochlear or respective views Cochlear as an organization may have.
